# Methamphetamine functions as a novel CD4^+^ T-cell activator via the sigma-1 receptor to enhance HIV-1 infection

**DOI:** 10.1038/s41598-018-35757-x

**Published:** 2019-01-30

**Authors:** Anil Prasad, Rutuja Kulkarni, Ashutosh Shrivastava, Shuxian Jiang, Kaycie Lawson, Jerome E. Groopman

**Affiliations:** 1 Division of Experimental Medicine, Beth Israel Deaconess Medical Center, Harvard Medical School, Boston, MA 02215 USA; 2 Molecular Biology Unit, Center for Advance Research, King George’s Medical University, Lucknow, India

## Abstract

Methamphetamine (Meth) exacerbates HIV-1 pathobiology by increasing virus transmission and replication and accelerating clinical progression to AIDS. Meth has been shown to alter the expression of HIV-1 co-receptors and impair intrinsic resistance mechanisms of immune cells. However, the exact molecular mechanisms involved in augmenting HIV-1 replication in T-cells are still not yet clear. Here, we demonstrate that pretreatment with Meth of CD4^+^ T-cells enhanced HIV-1 replication. We observed upregulation of CD4^+^ T-cell activation markers and enhanced expression of miR-34c-5p and miR-155 in these cells. Further, we noted activation of the sigma-1 receptor and enhanced intracellular Ca^2+^ concentration and cAMP release in CD4^+^ T-cells upon Meth treatment, which resulted in increased phosphorylation and nuclear translocation of transcription factors NFκB, CREB, and NFAT1. Increased gene expression of IL-4 and IL-10 was also observed in Meth treated CD4^+^ T-cells. Moreover, proteasomal degradation of Ago1 occurred upon Meth treatment, further substantiating the drug as an activator of T-cells. Taken together, these findings show a previously unreported mechanism whereby Meth functions as a novel T-cell activator via the sigma-1 signaling pathway, enhancing replication of HIV-1 with expression of miR-34c-5p, and transcriptional activation of NFκB, CREB and NFAT1.

## Introduction

Methamphetamine (Meth) abuse poses a daunting challenge in the prevention and treatment of HIV-1 infection^1^. Worldwide, Meth is the second most frequently used illicit drug^2^; its recreational popularity is one of the fastest-growing problems in the United States, as it enhances high-risk sexual behaviors and increases HIV-1 transmission^3–5^. Meth may also contribute to increased viral replication, accelerated progression to AIDS, poor adherence to anti-HIV-therapy and acquiring resistance to antiviral agents^6–9^. However, the exact molecular mechanisms of how Meth may enhance HIV-1 pathobiology and disease progression are yet to be fully elucidated.

Studies in animal models have shown that Meth treatment can increase viral load in HIV-1 infected animals^10,11^. In particular, Marcondes *et al*. reported that chronic Meth treatment raised viral load in the brains of Simian Immunodeficiency Virus (SIV) infected rhesus macaques, and resulted in activation of natural killer cells^10^. Clinical studies have shown that Meth can increase viral load in infected individuals, and its use also correlates with decreased efficacy of antiretroviral drugs^6,7,10,11^. *In vitro*, it has been demonstrated that Meth promotes HIV-1 replication in HIV-1 infected CD4^+^ T-cells and monocytes^11^. Further, Meth has been shown to enhance HIV-1 infection in dendritic cells (DCs) and macrophages by regulating the expression of HIV-1 co-receptors and DC-SIGN^12,13^. In microglial cells, Meth enhanced HIV-1 transcription via NFκB/SP1 dependent activation of HIV-1 LTR^14^. Conversely, Mantri *et al*. demonstrated inhibition of HIV-1 replication in the presence of Meth in human CD4^+^ T-cells and claimed that Meth modulates anti-HIV-1 miRNA expression in these cells^15^. Some recent reports have also indicated a role for Meth in inducing T-cell dysfunction and altering cell cycle progression, which can modulate the outcome of viral diseases^16,17^. Thus, although many studies have indicated a role for Meth in exacerbation of HIV-1 infection, the full range of effects of Meth on HIV-1 replication in CD4^+^ T-cells is still not yet clear.

In the brain, Meth has been shown to activate the dopamine and sigma-1 receptors (σ1-R)^18,19^. Moreover, Meth increased the expression of the σ1-R in astrocytes by activating Src, ERK mitogen-activated protein kinase, NFκB and cyclic-AMP response element-binding protein (CREB) pathways^19^. In addition, Meth can activate G-protein coupled receptors such as trace amine associated receptor 1 (TAAR1), and increase intracellular cyclic adenosine monophosphate (cAMP) levels^20^. Activation of these signaling pathways has been implicated in Meth mediated neuroinflammation^21^.

Meth also has been shown to modulate intracellular restriction factors by regulating miRNA expression, thereby enhancing HIV-1 infection^22^. Studies indicate an extensive alteration in miRNA expression in the brain of Meth-sensitized mice with post-translational regulation of gene expression^23^. In humans, miRNAs can be transcribed to primary miRNA by RNA polymerase II^24^. These primary miRNAs are further cleaved by RNAse III enzyme, Drosha, and its cofactor to generate short, stem-loop structured pre-miRNAs that are then exported to the cytoplasm where they are further processed to mature miRNA by Dicer1^25–27^. The mature miRNAs bind to a member of the Argonaute (AGO) family within a multicomponent complex known as RISC (RNA induced silencing complex), which functions either by miRNA-dependent mRNA decay or suppression of translation processes^25–27^. A significant downregulation of Ago2 was observed in the nucleus accumbens of Meth sensitized mice and expression of precursor miRNA was unaltered in these tissues, indicating that Meth may inhibit the Ago protein mediated splicing of precursor miRNA and its maturation^23^.

Here, we aimed to identify mechanisms that govern Meth enhanced HIV-1 infection of CD4^+^ T-cells. Specifically, we focused on pathways that have been found to be altered in other cell types concurrent with Meth exposure. We found that the sigma-1 receptor mediates Meth activation of transcription factors NFκB, CREB and NFAT1, known to facilitate replication of HIV-1. Further, we demonstrate that Meth treatment enhances HIV-1 replication in unstimulated CD4^+^ T-cells by inducing an activated phenotype via the sigma-1 receptor, characterized by cell surface markers and increased expression of miR-34c-5p and miR-155, which have also been shown to regulate HIV-1 infection. This occurred in conjunction with proteasomal degradation of Ago1, resulting in the loss of structural integrity of P-bodies. These studies reveal several novel mechanisms whereby Meth acts to impair immune defenses and augment HIV-1 infection in CD4^+^ T-cells.

## Results

### Meth pretreatment enhanced HIV-1 replication in CD4^+^ T-cells

**Figure 1:**
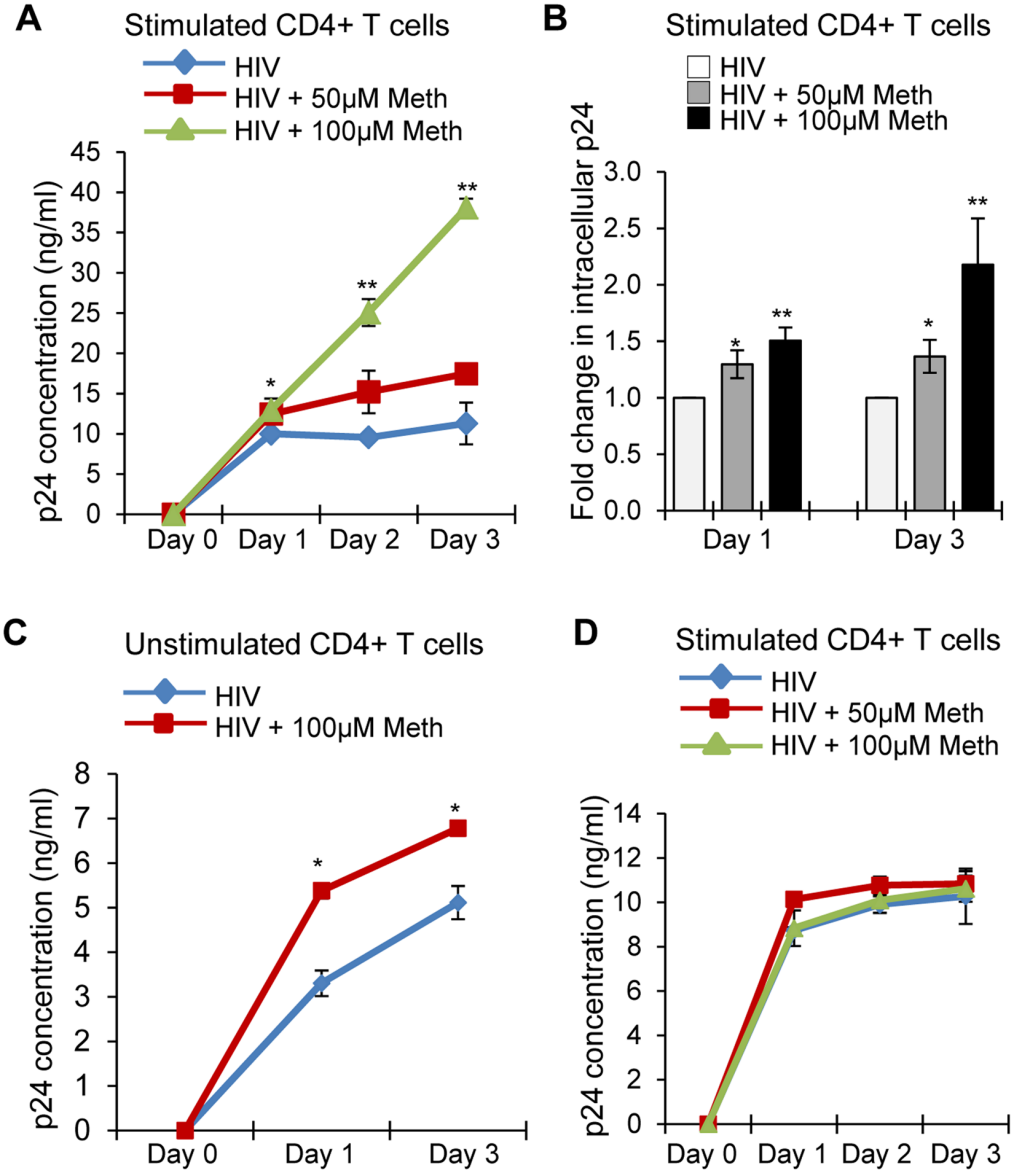
Meth pretreatment enhances HIV-1 replication in CD4^+^ T-cells. (**A**) PHA/IL-2 stimulated CD4^+^ T-cells were pretreated with or without Meth (50 or 100 µM) for 24 hours followed by incubation with HIV-1 BaL. Supernatants were harvested on days 0–3 and analyzed for extracellular HIV-1 p24 concentrations by ELISA (*p ≤ 0.05, **p ≤ 0.01). (**B**) CD4^+^ T-cells treated and infected in (A) were fixed, permeabilized, stained with FITC-conjugated p24 antibody, and intracellular p24 levels were analyzed by flow cytometry. Fold change was calculated by considering untreated cells as 1(*p ≤ 0.05, **p ≤ 0.01). (**C**) Unstimulated CD4^+^ T-cells (without PHA/IL-2) were pretreated with or without Meth (100 µM) for 24 hours followed by incubation with HIV-1 BaL. Supernatants were harvested on days 0–3 and analyzed for extracellular HIV-1 p24 concentrations by ELISA (*p ≤ 0.05). (**D**) Stimulated CD4^+^ T-cells were treated with or without Meth (50 or 100 µM) and incubated with HIV-1 BaL simultaneously. Supernatants were harvested on days 0–3 and analyzed for extracellular HIV-1 p24 concentrations by ELISA. Data represent the mean ± SEM of 3 experiments done in triplicate for untreated cells vs. cells treated with Meth.

Previous studies in *in vitro* models have demonstrated that Meth enhances HIV-1 replication in T-cells, DCs, macrophages and neural progenitor cells^11–14^. The significance of these results is supported by an epidemiological study, which demonstrated increased viral loads in Meth using HIV-1 infected individuals compared with non-users who were infected^28^. However, the effects of Meth on HIV-1 replication in CD4^+^ T-cells are controversial, as Mantri *et al*. reported inhibition of HIV-1 replication in Meth treated CD4^+^ T-cells^15^. Here, we first examined HIV-1 BaL replication in PHA/IL-2 stimulated CD4^+^ T-cells (hereafter referred to as CD4^+^ T-cells) after either pretreating cells with 50 µM or 100 µM Meth for 24 hours and then infecting cells with HIV-1, or treating the cells with HIV-1 and Meth simultaneously. We analyzed HIV-1 p24 titer at different time points in the cell supernatants and observed significant increases in HIV-1 p24 titer in cells pretreated with 50 µM or 100 µM of Meth on days 1, 2 and 3 (Fig. 1A). We confirmed these results by estimating the intracellular HIV-1 p24 by flow cytometry, which showed a significant increase in HIV-1 p24 in cells pretreated with Meth compared to control cells (Fig. 1B). It has been shown that naïve CD4^+^ T-cells or resting T-cells are refractory to HIV-1 infection^29^. Notably, unstimulated CD4^+^ T-cells (without PHA/IL-2) exhibited a naïve phenotype, characterized by low expression of CD4^+^ T-cell activation markers CD69 and CD25, as well as higher expression of naïve CD4^+^ T-cell marker CD45RA^30^. Thus we next analyzed the effects of Meth pretreatment on HIV-1 replication in unstimulated CD4^+^ T-cells; we observed enhanced HIV-1 replication in these cells compared to untreated cells (Fig. 1C). Intriguingly, we did not observe any significant change in the HIV-1 p24 titer when CD4^+^ T-cells were treated with Meth and HIV-1 simultaneously (Fig. 1D). These results indicate that Meth may alter cellular conditions favorable for HIV-1 infection in both stimulated and unstimulated CD4^+^ T-cells.

### Meth enhanced intracellular calcium and cAMP levels in CD4^+^ T-cells

**Figure 2:**
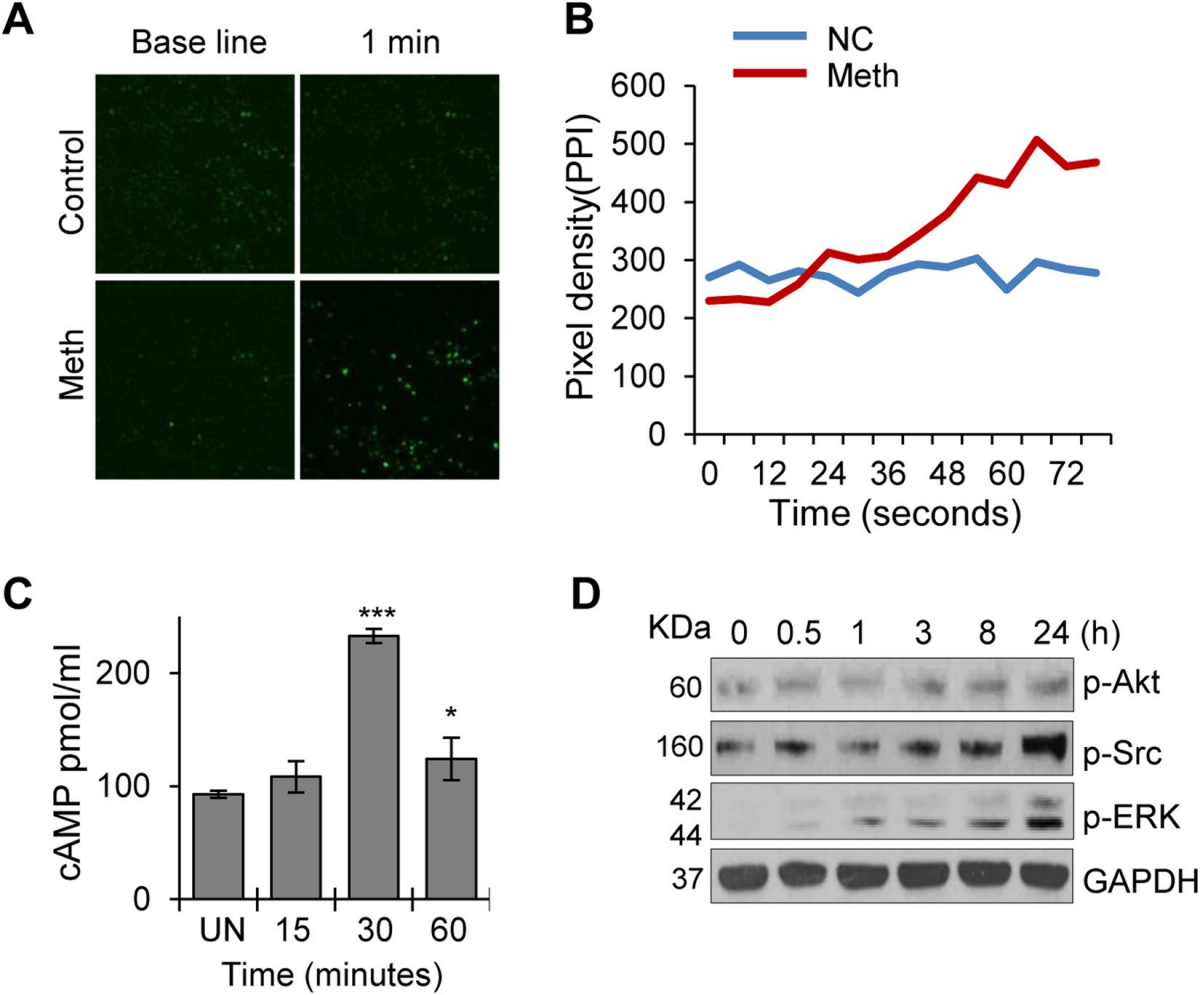
Meth enhances intracellular calcium and cAMP levels in CD4^+^ T-cells. (**A**) Calcium influx was measured by staining the CD4^+^ T-cells with Fluo-4,AM, followed by addition of 100 µM Meth and live cell imaging by confocal microscopy. (**B**) Pixel densities of images in (**A**) were determined by ImageJ and plotted against time (seconds). Data represent the mean ± SEM of 3 independent experiments for untreated cells vs. cells treated with Meth. (**C**) cAMP levels were analyzed by ELISA after treating CD4^+^ T-cells with 100 µM Meth and acquiring samples at different time points (0, 15 min, 30 min and 1 hour). Data represent the mean ± SD of 3 independent experiments (*p ≤ 0.05, ***p ≤ 0.001). (**D**) CD4^+^ T-cells were untreated or treated with 100 µM Meth for the indicated time points (0.5–24 hours), lysed, and protein extracts were analyzed by Western blotting for the signaling molecules p-Akt, p-Src and p-ERK. GAPDH was used as a loading control. Full-length blots are presented in Supplementary Fig. S1.

Meth has been shown to induce increased intracellular calcium concentration [Ca^2+^] in neuronal cells^30^. To further explore the molecular mechanisms involved in Meth mediated effects on CD4^+^ T-cells, we analyzed the release of second messengers Ca^2+^ and cAMP after treating the cells with the drug. We observed significant increases in intracellular calcium levels upon Meth treatment; the maximum concentration was observed at 1 minute. This result suggests that Meth may have released the calcium from intracellular stores (Fig. 2A,B). In addition, Meth has been shown to modulate cytokine secretion by regulating a cAMP/PKA/CREB signaling pathway in microglial cells^31^. We observed significant increases in cAMP at 30 minutes after Meth treatment indicating that Meth can activate cAMP mediated signaling pathways in CD4^+^ T-cells (Fig. 2C). Notably, pathways involving cAMP and transcription factors such as CREB have been associated with T-cell activation^32^. Western blot analysis also revealed increased phosphorylation of Akt, Src and ERK1/2 upon Meth treatment in these cells (Fig. 2D).

### Meth induces increased phosphorylation and nuclear translocation of CREB, NFAT1 and NFκB in CD4^+^ T-cells

**Figure 3:**
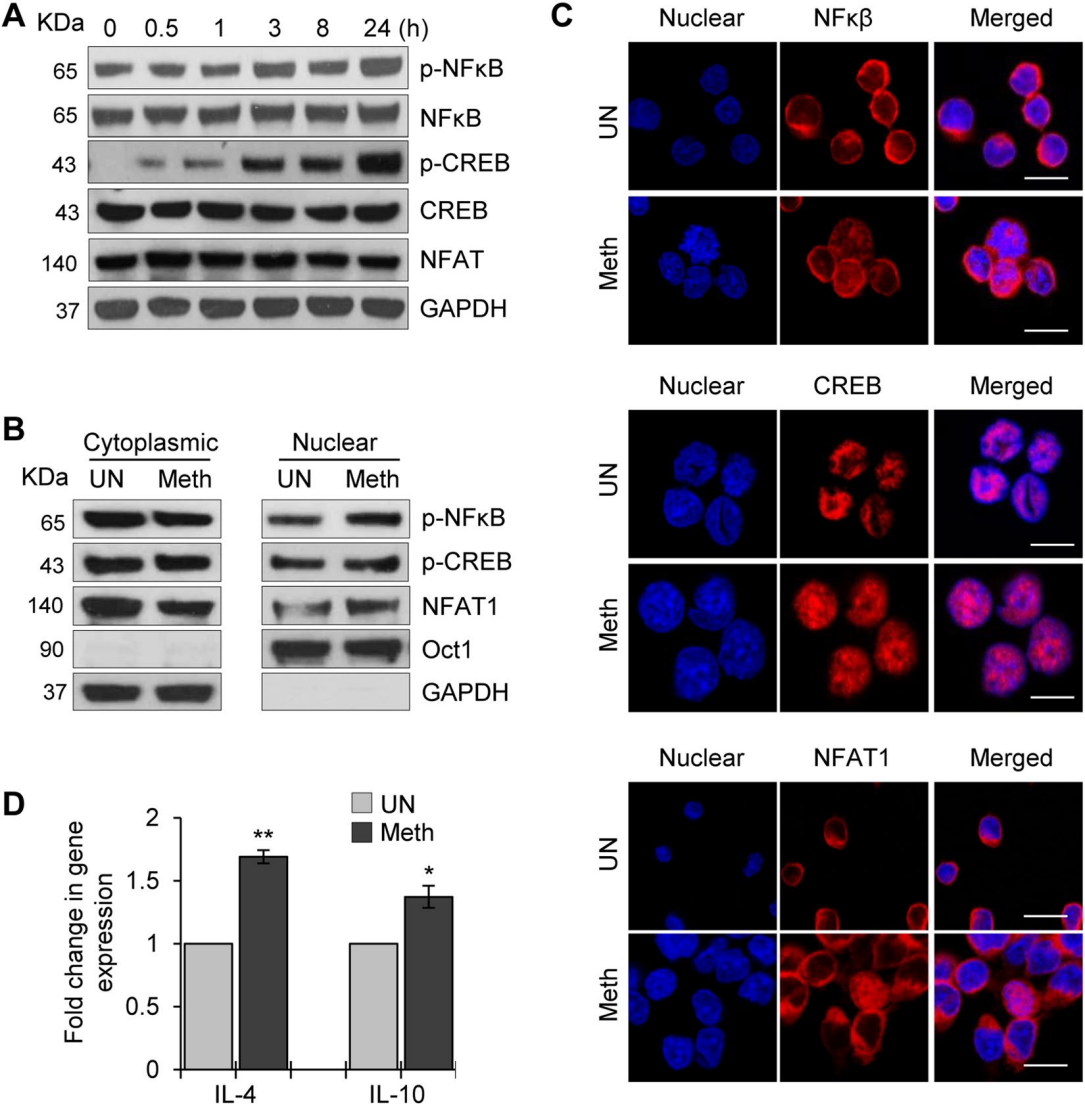
Meth induced increased phosphorylation and nuclear translocation of CREB, NFAT1 and NFκB in CD4^+^ T-cells. (**A**) CD4^+^ T-cells were untreated or treated with 100 µM Meth for different time points (0.5–24 hours), lysed and the protein extracts were analyzed for the activation of transcription factors NFκB and CREB and NFAT1 by Western blotting. GAPDH was used as a loading control. Full-length blots are presented in Supplementary Fig. S2. (**B**) CD4^+^ T-cells were untreated or treated with 100 µM Meth for 1 hour; cytoplasmic and nuclear extracts were isolated and analyzed for activated transcription factors. Oct-1 is nuclear loading control while GAPDH is cytoplasmic control. Full-length blots are presented in Supplementary Fig. S2. (**C**) Confocal images of NFκB (upper panel), CREB (middle panel) or NFAT1 (lower panel) staining in CD4^+^ T-cells untreated or treated with 100 µM Meth for 1 hour. Meth treated samples show nuclear translocation of NFκB, CREB and NFAT1. Scale bar = 10 µm. (**D**) CD4^+^ T-cells were untreated or treated with 100 µM Meth for 24 hours, RNA was isolated, and IL-4 and IL-10 gene expression was analyzed by qRT-PCR. Fold change was calculated by normalizing the Meth treated cells to untreated cells. Data represent the mean ± SD of 3 independent experiments (*p ≤ 0.05, **p ≤ 0.01).

We next analyzed the effects of Meth on the downstream cellular transcription factors, CREB, nuclear factor of activated T-cells (NFAT1), and NFκB, which are associated with T-cell immune activation^32^. We first examined the phosphorylation status and levels of expression of these molecules after treating CD4^+^ T-cells with Meth at various time points. Our analysis revealed increased phosphorylation of CREB and the p65 subunit of NFκB. However, total expression of these molecules was unaltered (Fig. 3A). Next, we analyzed the effects of Meth on their expression in nuclear and cytoplasmic fractions. We observed increased nuclear translocation of p-NFκB, p-CREB and NFAT1 in Meth treated cells compared to untreated cells, with levels of p-NFκB and NFAT1 significantly reduced in the cytoplasmic fractions (Fig. 3B). We performed confocal microscopy to confirm these results and observed increased nuclear translocation of p-NFκB, p-CREB and NFAT1 in Meth treated cells compared to control cells (Fig. 3C). These results indicate that Meth induced translocation of p-NFκB, p-CREB and NFAT1 from the cytoplasm to the nucleus in CD4^+^ T-cells.

NFAT1 has been shown to regulate the expression of cytokines IL-4 and IL-10 during T-cell activation^33,34^. qRT-PCR analysis revealed increased levels of mRNA of both IL-4 and IL-10 in Meth treated cells compared to control cells, suggesting that Meth induced translocation of NFAT1 may participate in the increased transcription of IL-4 and IL-10 in CD4^+^ T-cells (Fig. 3D).

### Meth mediated its effect through activating the sigma-1 receptor in CD4^+^ T-cells

**Figure 4:**
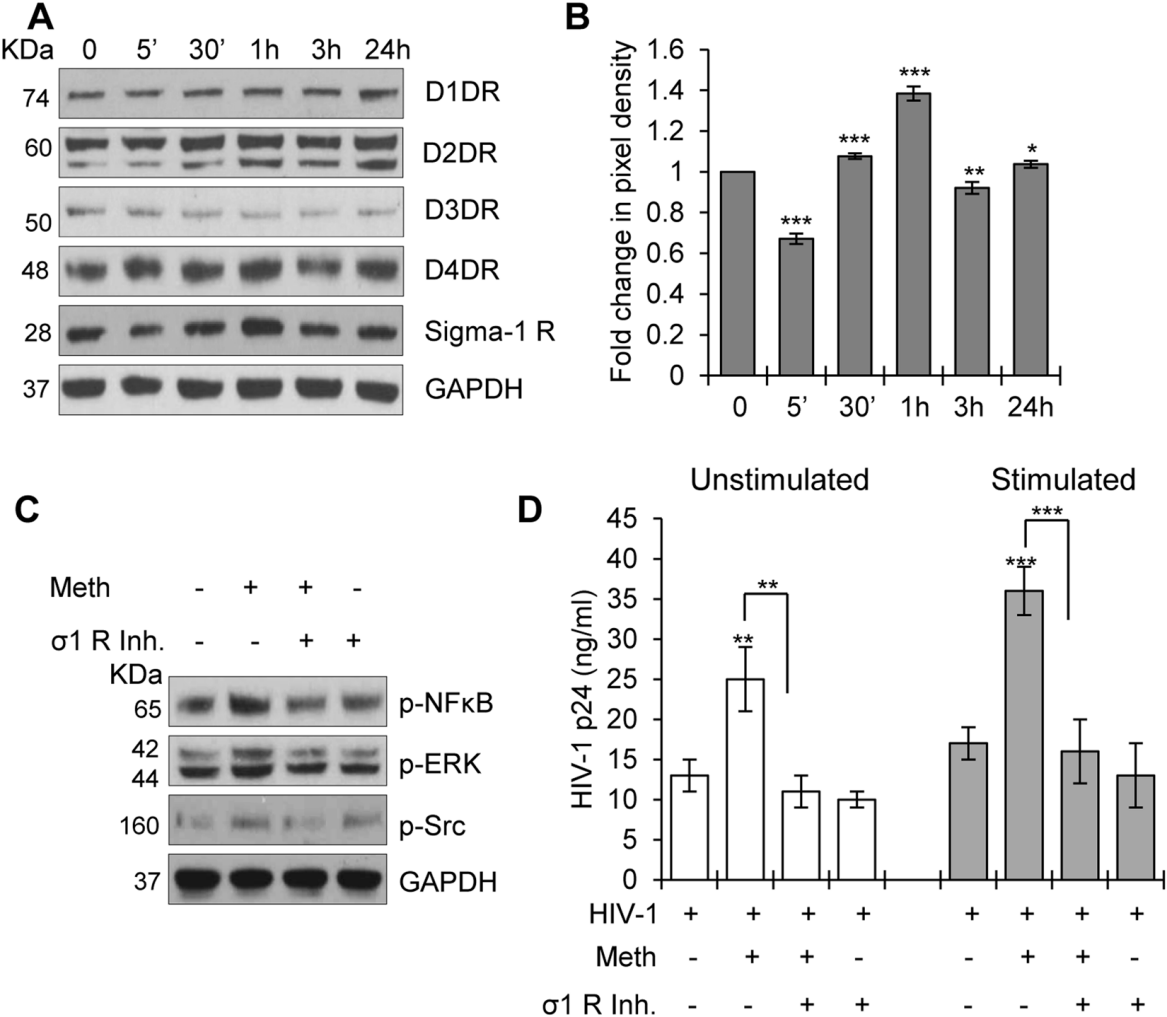
Effects of Meth on dopamine receptors and sigma-1 receptor in CD4^+^ T-cells. (**A**) CD4^+^ T-cells were untreated or treated with 100 µM Meth for different time points (5 mins-24 hours), lysed and the protein extracts were analyzed for the expression of various dopamine receptors and sigma-1 receptor. GAPDH used as a loading control. Full-length blots are presented in Supplementary Fig. S3. (**B**) Fold change in the pixel density of sigma-1 receptor expression in (**A**). All values normalized to untreated sample. (*p ≤ 0.05, **p ≤ 0.01, ***p ≤ 0.001). (**C**) CD4^+^ T-cells were untreated or treated with 10 µM sigma-1 receptor inhibitor (σ1 R inh.) for 1 hour, then treated with or without 100 µM Meth for 1 hour, followed by lysis and analysis of protein extracts for the indicated activated signaling molecules by Western blotting. GAPDH used as a loading control. Full-length blots are presented in Supplementary Fig. S3. (**D**) HIV-1 p24 titer on day 3 after HIV-1 infection in unstimulated and stimulated CD4^+^ T-cells pretreated with or without sigma-1 receptor inhibitor (σ1 R inh.) and treated in the presence or absence of Meth. Data represent the mean ± SD of 3 independent experiments (**p ≤ 0.01, ***p ≤ 0.001).

Meth has been shown to exert its effects by activating dopamine receptors and/or sigma-1 receptors in the brain^18,19^. Hence, we analyzed effects of Meth on these receptors in CD4^+^ T-cells. All four dopamine receptors (D1DR, D2DR, D3DR, and D4DR) and the sigma-1 receptor were expressed constitutively, and we observed significant increased expression only of the sigma-1 receptor after Meth treatment (Fig. 4A). These observations indicated that Meth may act via the sigma-1 receptor in CD4^+^ T-cells (Fig. 4A,B). To confirm its functional role, we treated the cells with sigma-1 receptor specific inhibitor (Dihydrobromide, BD1047), then with Meth, and analyzed the activation of downstream signaling molecules by Western blot analysis. The sigma-1 receptor inhibitor significantly blocked the phosphorylation of NFκB, ERK and Src kinases (Fig. 4C). These results indicate that Meth induces the activation of transcription factors in CD4^+^ T-cells by activating the sigma-1 receptor. Next, to confirm the role of sigma-1 receptor in Meth mediated enhanced HIV-1 replication in CD4^+^ T-cells, we employed the sigma-1 receptor inhibitor in both unstimulated and stimulated cells. We found that this inhibitor significantly abrogated Meth mediated enhanced HIV-1 replication in both the stimulated and unstimulated cell types (Fig. 4D). These results confirm that sigma-1 receptor signaling is needed for Meth mediated enhanced HIV-1 replication in CD4^+^ T-cells.

### Meth enhanced expression of miR-34c-5p and miR-155 and induced abnormal activation of CD4^+^ T-cells

**Figure 5:**
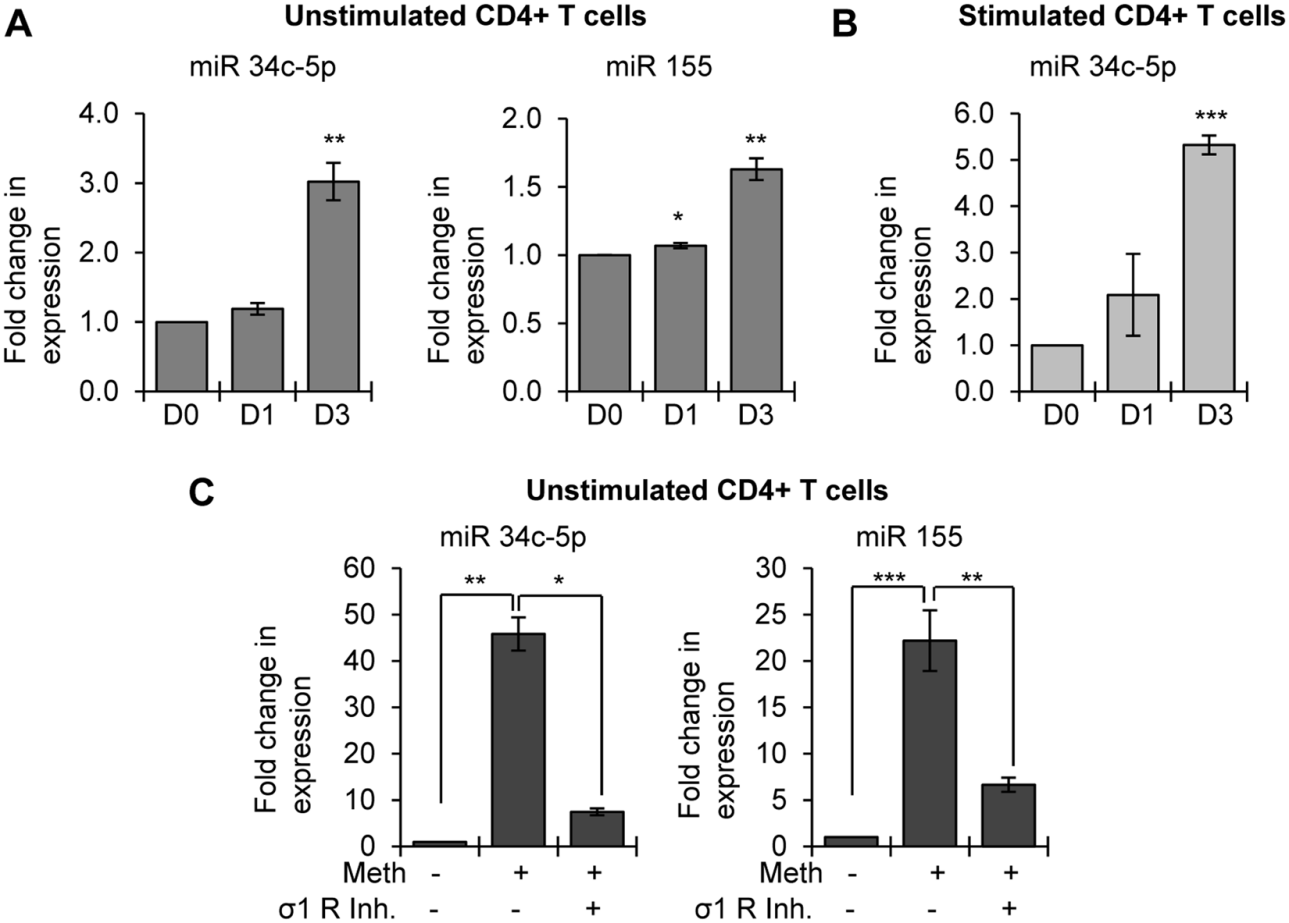
Analysis of miRs-34c-5p and 155 in Meth treated CD4^+^ T-cells: Unstimulated (**A**) and Stimulated (**B**) CD4^+^ T-cells were treated with Meth (100 µM) for 3 days, cells were harvested on days 0, 1 and 3 (D0, D1 and D3), RNA was isolated, and the expression of miRs-34c-5p and 155 was analyzed by qRT-PCR by normalizing all the samples to untreated samples (*p ≤ 0.05, **p ≤ 0.01, ***p ≤ 0.001). (**C**) Expression analyses of miRs-34c-5p and 155 by qRT-PCR in untreated and Meth treated unstimulated CD4^+^ T-cells in the presence or absence of sigma-1 receptor inhibitor (σ1 R inh.) at day 3 (*p ≤ 0.05, **p ≤ 0.01, ***p ≤ 0.001).

Previous studies have shown that miR-34c-5p can activate naïve CD4^+^ T-cells by TCR stimulation and thereby make cells more susceptible to HIV-1 infection^35^. In addition, miR-155 has been shown to be upregulated in activated T-cells^35^. Since we observed increased HIV-1 infectivity after Meth pretreatment, we examined whether Meth treatment altered the expression of these two miRNAs in unstimulated CD4^+^ T-cells, thus driving them toward an activated state. Interestingly, we found that the expression of both miR-34c-5p and miR-155 increased in unstimulated CD4^+^ T-cells by more than 3 fold and 1.5 fold respectively after drug treatment (Fig. 5A). The increase we observed in these two miRNAs, which have been associated with T-cell activation, further indicates that Meth-treatment facilitates CD4^+^ T-cell activation. Next, we analyzed expression of miR-34c-5p expression in PHA/IL-2 stimulated CD4^+^ T-cells upon treating with Meth. We also found enhanced expression of this miRNA in drug treated activated cells compared to control cells (Fig. 5B). These results indicate that Meth induced increased expression of miR-34c-5p may further predispose CD4^+^ T-cells to HIV-1 replication. To explore a possible mechanism for increased miR-34c-5p and miR-155 expression, we tested their abundance with or without the sigma-1 receptor inhibitor; this also allowed us to analyze the role of Meth mediated activation of the sigma-1 receptor. We observed that the increased expression of these two miRNAs due to Meth was significantly abrogated in the presence of the inhibitor (Fig. 5C). These results indicate that Meth induces the expression of miR-34c-5p and miR-155 via activation of the sigma-1 receptor.

**Figure 6:**
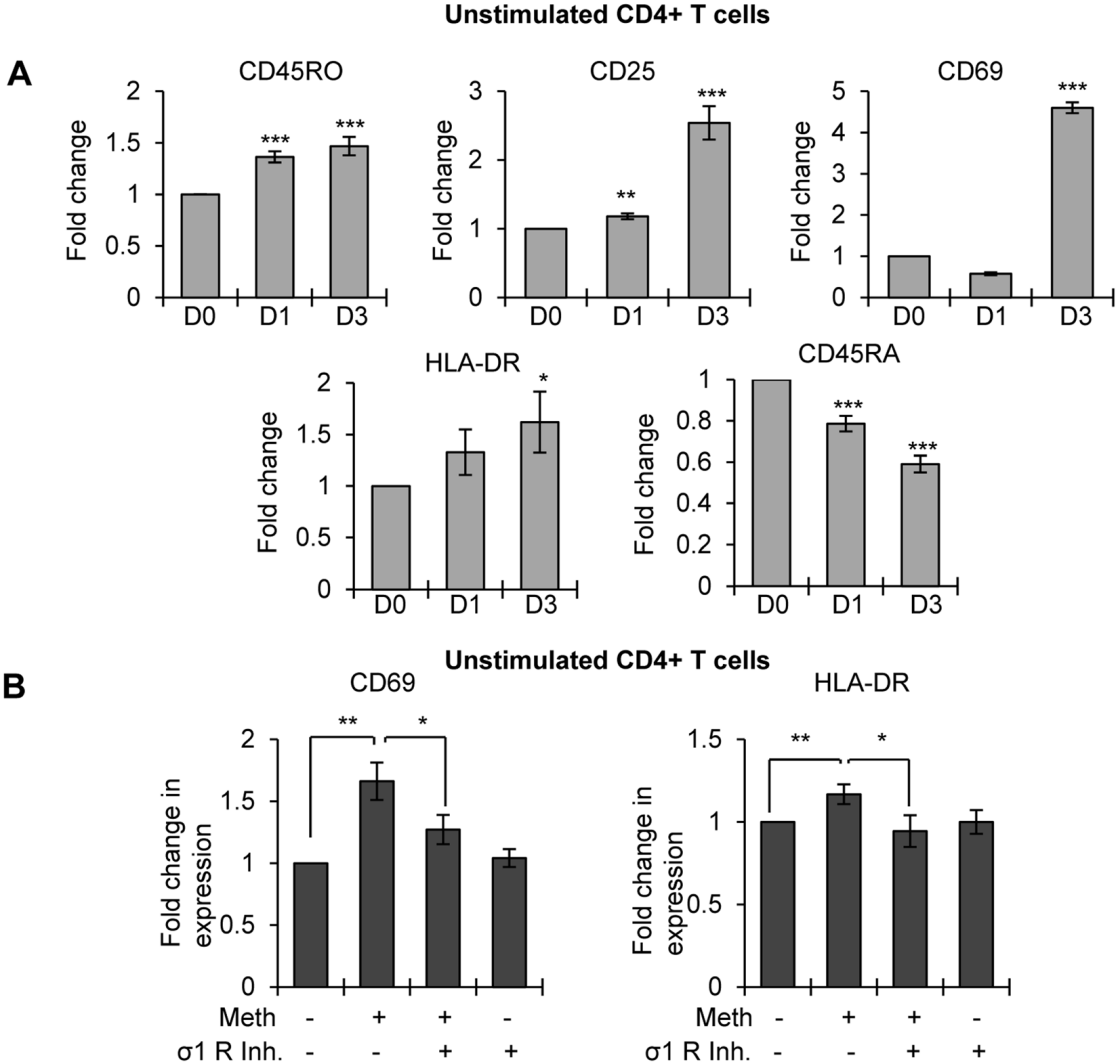
Meth induced the expression of activation markers in unstimulated CD4^+^ T-cells: (**A**) Unstimulated CD4^+^ T-cells were treated with or without Meth (100 µM) for 3 days, cells were harvested on days 0, 1 and 3 (D0, D1 and D3), stained for the indicated T-cell activation markers, fixed and analyzed by flow cytometry. Bar diagrams showing the fold change in the expression of indicated activation markers in Meth treated unstimulated CD4^+^ T-cells. Fold change was calculated by normalizing the Meth treated cells to untreated cells (*p ≤ 0.05, **p ≤ 0.01, ***p ≤ 0.001). (**B**) Fold change in the expression of CD69 (left panel) and HLA-DR (right panel) in untreated or Meth treated unstimulated CD4^+^ T-cells in the presence or absence of sigma-1 receptor inhibitor (σ1 R inh.) after 3 days (*p ≤ 0.05, **p ≤ 0.01).

Next, to analyze the effects of Meth mediated activation of signaling molecules and alteration of miRNA expression on T-cell activation, we tested the expression levels of CD4^+^ T-cell activation markers by flow cytometry after treating unstimulated CD4^+^ T-cells with Meth at different time points. Drug treatment modestly increased CD45RO expression (1.3 fold) and HLA-DR (1.5 fold). Intriguingly, we observed significant increases in the expression of other CD4^+^ T-cell activation markers such as CD69 (>4.5 fold) and CD25 (>2.5 fold). Naïve T-cells have a CD45RA^+^ phenotype, which we found to be decreased in unstimulated CD4^+^ T-cells by 40% on day 3 of Meth treatment (Fig. 6A). Thus, taken together, Meth treatment can elevate the expression of miRs-34c-5p and 155 which have been shown to increase CD4^+^ T-cell activation, and may make the cells more susceptible to HIV-1 infection. We then analyzed the role of Meth mediated activation of the sigma-1 receptor in enhanced expression of T-cell activation markers in unstimulated CD4^+^ T-cells using sigma-1 receptor inhibitor. We observed that drug mediated increased expression of CD69 and HLA-DR was significantly abrogated in the presence of the sigma-1 receptor inhibitor (Fig. 6B). These studies further indicate that Meth induces a novel pathway of CD4^+^ T-cell activation by triggering sigma-1 receptor signaling.

### Meth induced degradation of Ago1 and altered structural integrity of P-bodies

**Figure 7:**
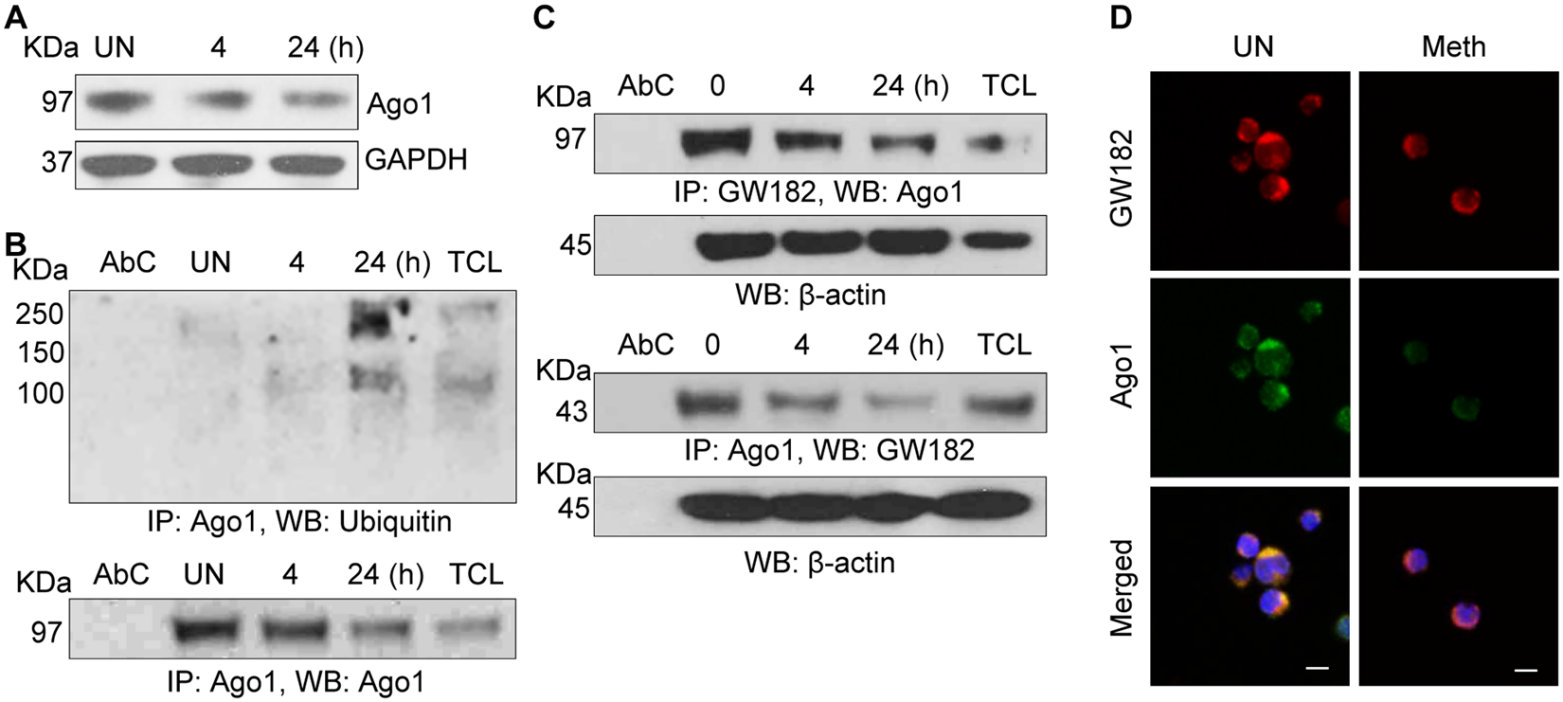
Meth induced degradation of Ago1 and altered structural integrity of P-bodies: (**A**) CD4^+^ T-cells were untreated or treated with Meth (100 µM) for 0, 4 and 24 hours, lysed and Ago1 expression was analyzed by Western blotting. GAPDH used as a loading control. Full-length blots are presented in Supplementary Fig. S4 (**B**) CD4^+^ T-cell lysates in (**A**) were immunoprecipitated with Ago1 antibody and subjected to Western blot analysis using Ubiquitin antibody. Ago1 served as a loading control; AbC = Antibody control, TCL = Total cell lysate. Results are representative of 3 independent experiments. (**C**) CD4^+^ T-cell lysates in (A) were immunoprecipitated with GW182 antibody (upper panel) or Ago1 antibody (lower panel) and subjected to Western blot analysis using Ago1 (upper panel) or GW182 (lower panel) antibodies. B-Actin served as a loading control; AbC = Antibody control, TCL = Total cell lysate. Results are representative of 3 independent experiments. Full-length blots are presented in Supplementary Fig. S4 (**D**) Confocal images of GW182 and Ago1 interaction in CD4^+^ T-cells, untreated or treated with Meth (100 µM) for 24 hours. Scale bar = 10 µm. Results are representative of 3 independent experiments.

A recent study has revealed that T-cell activation induces global post-transcriptional miRNA down-regulation and proteasomal degradation of Argonaute (Ago) proteins^36^. Ago proteins are altered upon T-cell activation, and are known to play a central role in RNA-silencing process and are critical for miRNA mediated translational repression and miRNA-mediated mRNA degradation^25,37^. Hence, we analyzed the effects of Meth treatment on Ago1 expression in CD4^+^ T-cells. We found that Meth treatment significantly inhibited Ago1 expression within 24 hours after treatment, which is consistent with T-cell activation (Fig. 7A). Further, we analyzed the mechanisms involved in Meth mediated inhibition of Ago1 expression; we found increased ubiquitination of Ago1 in drug treated cells compared to untreated cells (Fig. 7B). Moreover, the level of Ago1 mRNA was unaltered in Meth treated cells (data not shown). These results indicate that Meth treatment may induce proteasomal degradation of Ago1 in CD4^+^ T-cells as a result of induced activation. In addition, we also found that mRNA expression levels of Drosha and Dicer were unaltered in Meth treated CD4^+^ T-cells (data not shown).

Processing bodies (P-bodies) are important, discrete foci in the cytoplasm that play a key role in mRNA gene silencing and regulation of mRNA turnover^38,39^. Ago proteins and GW182 are the main components of P-bodies^38–40^. Since we observed decreased expression of Ago1 in Meth treated CD4^+^ T-cells, we next analyzed the structural integrity of P-bodies in Meth treated CD4^+^ T-cells. Confocal microscopic analysis and immunoprecipitation analysis showed decreased co-localization of Ago1 and GW182 in Meth treated cells compared to control cells (Fig. 7C,D). These results indicate that Meth may alter the structural integrity of P-bodies in CD4^+^ T-cells.

## Discussion

Meth use is associated with a high risk of contracting HIV-1 infection, and its transmission^1–5^. Moreover, Meth enhances the viral load in HIV-1 positive, active drug using individuals by increasing viral replication in T-cells, monocytes and neural progenitor cells^7,11^. However, the molecular mechanisms involved in these Meth mediated effects in the immunopathogenesis of HIV-1 still are not fully elucidated. Here, we characterized novel molecular effects of Meth on CD4^+^ T-cells and their implications in HIV-1 replication.

Our experiments reveal that pretreatment of CD4^+^ T-cells with Meth significantly enhanced HIV-1 replication; however, there was no significant change in replication when cells were treated with HIV-1 and Meth simultaneously. A previous study by Skowronska demonstrated enhanced HIV-1 replication in neural progenitor cells when they were pretreated with Meth but not when they are exposed to HIV-1 and Meth together^14^. To address the mechanism of this effect, they point to Meth mediated NFκB/SP1 dependent activation of the HIV-1 LTR^14^. Intriguingly, our experiments with unstimulated CD4^+^ T-cells also showed that Meth pretreatment enhanced HIV-1 replication. *In vitro*, single cell cultures of resting or naïve CD4^+^ T-cells are resistant to HIV-1 infection^41,42^. However, *in vivo* the tissue microenvironment facilitates the activation of naïve T-cells and provides conditions favorable for productive HIV-1 infection^41–43^. Hence, CD4^+^ T-cell activation is considered to be a key factor that facilitates infection^44,45^. Moreover, expression of the T-cell activation markers CD25 and HLA-DR has been shown to correlate with enhanced HIV-1 infection^43^. When we analyzed cell activation markers in unstimulated CD4^+^ T-cells upon Meth treatment, we observed significant increases in CD25 and HLA-DR. We also observed increased expression of the activation markers CD69 and CD45RO, and a modest decline in the naïve CD4^+^ T-cell marker CD45RA. In addition, after Meth treatment of unstimulated CD4^+^ T-cells, we observed significant increases in the expression of miR-34c and miR-155. Transcriptional upregulation of miR-34c has been shown to occur during activation of CD4^+^ T-cells. Further, both of these miRNAs are reported to promote HIV-1 replication in CD4^+^ T-cells^35^.These findings indicate that Meth can act as an activator of CD4^+^ T-cells which could contribute to enhanced HIV-1 infection. Our finding corresponds to a clinical study by Massanella *et al*., where they observed enhanced CD4^+^ and CD8^+^ T-cell proliferation, and CD4^+^ T-cell activation and exhaustion in Meth using HIV-1-infected ART-suppressed individuals^16^.

Our study showed that Meth significantly increased intracellular [Ca^2+^] and cAMP levels in CD4^+^ T-cells. Meth is known to induce increased cytosolic calcium concentration flux and cAMP release in neuronal cells^20,31^. Both of these pathways play major roles in T-cell signaling and activation, and are involved in downstream regulation of the transcription factors NFκB, CREB and NFAT1^32^. Interestingly, the NFκB/SP1 complex has been shown to mediate enhanced HIV-1 transcription in T-cells^14^. In addition, CREB has been shown to play a role in Meth addiction and activation of neuroinflammatory signaling pathways^21^. We observed increased phosphorylation and enhanced nuclear translocation of these transcription factors in Meth treated CD4^+^ T-cells. NFAT1 is a positive regulator of gene transcription of several cytokines including IL-4 and IL-10^33,34^. Consistent with these studies, we observed enhanced IL-4 and IL-10 gene transcription in Meth treated CD4^+^ T-cells. From our findings, we infer that Meth induces activation of Ca^2+^ signaling and cAMP pathways which may be involved in the abnormal activation and regulation of cytokine gene expression in CD4^+^ T-cells.

Meth induced CD4^+^ T-cell activation via the sigma-1 receptor. Involvement of the sigma-1 receptor was also implicated in Meth mediated activation of astrocytes via Src, ERK/ mitogen-activated protein kinase, and CREB pathways^19^. Indeed, in our study we found Meth treatment similarly led to the activation of these downstream signaling pathways. Furthermore, enhanced nuclear translocation of CREB may have interacted with the promoter of the sigma-1 receptor resulting in the enhanced expression of the sigma-1 receptor in CD4^+^ T-cells.

MiRNAs are implicated in various biological processes including drug addiction and as modulators of post-transcriptional gene expression^23,46,47^. Recently, Liu *et al*. reported significant downregulation of Ago2, indicating that miRNA downregulation may be due to inhibition of Ago2 mediated splicing of precursor miRNA and not due to inhibition at the transcriptional level^23^. Consistent with this study, we observed proteasomal degradation of Ago1, that may have resulted in altered structural integrity of P-bodies, which play key roles in silencing of target mRNAs. Interestingly, prior studies have shown that during T-cell activation, Ago proteins undergo proteasome mediated degradation^36^. This further confirms Meth as a novel activator of CD4^+^ T-cells.

In sum, we elucidate several mechanisms through which Meth can enhance HIV-1 replication in CD4^+^ T-cells, and act as a novel activator of CD4^+^ T-cells through upregulation of miR-34c-5p and increased expression of several CD4^+^ T-cell activation markers. Consistent with this new insight into the drug as a T-cell activator, we found that Meth alone and in combination with HIV-1 infection induced extensive Ago1 degradation, which can result in loss of structural integrity of P-bodies and formation of miRNA induced silencing complexes, and has been linked to decreased anti-viral response and increased HIV-1 transcription^48^. We also show that Meth mediated activation of T-cells resulted in activation of the transcription factors NFκB, CREB and NFAT1, which may contribute to increased HIV-1 replication and production of inflammatory cytokines. Thus, multiple molecular mechanisms emanating from Meth as a T-cell activator likely contribute to enhanced HIV-1 pathogenesis in drug using individuals and provide insights into the development of novel therapeutic strategies to limit HIV-1 in these individuals.

## Materials and Methods

### Cells, HIV-1 and constructs

Buffy coats were obtained from the Blood Transfusion Service, Massachusetts General Hospital, Boston, MA, in compliance with the Beth Israel Deaconess Medical Center Committee on Clinical Investigations (CCI) protocol #2008-P-000418/5. Buffy coats were provided at this institution for research purposes; therefore, no informed consent was further needed. In addition, buffy coats were provided without identifiers. This study was approved by Beth Israel Deaconess Medical Center’s CCI, Institutional Review Board, and Privacy Board appointed to review research involving human subjects. The experimental procedures were carried out in strict accordance with approved guidelines.

Human peripheral blood mononuclear cells were isolated from buffy coats and CD4^+^T cells were isolated using a negative selection kit respectively, per manufacturer’s protocol (STEMCELL Technologies, Inc.). For PHA/IL-2 stimulated CD4^+^ T-cells, CD4^+^ T-cells were cultured in complete RPMI culture medium supplemented with PHA-L (1 μg/ml) and IL-2 (10 ng/ml) (PeproTech, Rocky Hill, NJ) at 2 × 10^6^ cells/ml for 3 days. For unstimulated CD4^+^ T-cells, CD4^+^ T-cells were cultured in complete RPMI culture medium without PHA and IL-2. Purity of these T cells was analyzed using CD3 and CD4 staining and flow cytometry.

### Antibodies and reagents

HIV-1 BaL was obtained from the NIH AIDS Research and Reference Reagent Program, National Institute of Allergy and Infectious Diseases, NIH. The HIV-1 BaL strain has been shown to efficiently infect, via the CCR5 co-receptor, and replicate within CD4^+^ T-cells^49^. Argonaute-1, p-NFκB, NFκB, p-ERK, p-Src, p-Akt, NFAT1, p-CREB, CREB, Oct-1, Ubiquitin, and β-Actin antibodies were obtained from Cell Signaling Technology (Danvers, MA). GW182, D1DR, D2DR, D3DR, D4DR, Sigma-1 Receptor, and GAPDH antibodies were obtained from Santa Cruz Biotechnology, Inc. (Santa Cruz, CA). FITC-conjugated p24 GAG (6604665) antibody was obtained from Beckman Coulter, Inc. (Brea, CA). PE-conjugated CD45RO, PE-conjugated HLA-DR, FITC-conjugated CD45RA, FITC-conjugated CD25, FITC-conjugated CD69, PE isotype control, and FITC isotype control antibodies were purchased from Biolegend (San Diego, CA). p-NFAT1 antibody was obtained from Thermo Fisher Scientific (Waltham, MA). Fluo-4, AM was purchased from Invitrogen (Carlsbad, CA). Methamphetamine hydrochloride and sigma-1 receptor inhibitor (BD1047) were purchased from Sigma Aldrich (St. Louis, MO).

### HIV-1 replication assay

CD4^+^ T-cells cultured at 2 × 10^6^/ml were untreated (control cells) or pre-treated with 50 μM or 100 μM Meth (dissolved in water) at 37 °C for 24 hours then incubated with HIV-1 BaL (10 ng/ml, p24) at 37 °C for 3 days. Culture supernatants were harvested on days 0, 1, 2 and 3 and viral replication was assessed by quantitating the p24 in the supernatants by ELISA, whereby increasing p24 concentration across time points indicates that HIV replication has occurred. This technique has been well established as a measure of HIV replication *in vitro* and *in vivo*^50^.

### Flow cytometric analyses

CD4^+^ T cells, isolated as aforementioned, were cultured in complete medium without PHA and IL-2 but were treated with or without 100 µM Meth for 3 days. Cells were harvested on days 0, 1 and 3, stained with the T-cell activation markers, and analyzed by flow cytometry. CD4^+^ T cells were stained with the marker antibodies conjugated with fluorophores or with their respective isotypes. The positively stained cells were gated based off the respective isotype.

Briefly, cell surface staining was performed by washing cells in 0.5% BSA in 1X PBS followed by incubation with fluorescent antibodies. Cells were fixed in 10% formalin with 4% formaldehyde (Sigma Aldrich, St. Louis, MO) for 30 minutes before washing twice more with 0.5% BSA in 1X PBS. Cells were analyzed in 1X PBS solution. Intracellular p24 was analyzed by staining the cells using FITC-conjugated p24 GAG antibody and analyzed on BD LSRII (BD Biosciences, Franklin Lakes, NJ). For p24 intracellular staining, the cells were stained with anti-gag antibody conjugated to FITC or FITC isotype control. The FITC positive cell population was gated based off the isotype control. Intracellular staining was performed by first washing cells in 0.5% BSA in 1X PBS. Then, cells were fixed in 10% formalin with 4% formaldehyde (Sigma Aldrich, St. Louis, MO) for 30 minutes before washing twice with 0.5% BSA in 1X PBS. Cells were permeabilized in 1X BD FACS^TM^ Permeabilizing Solution 2 (BD Biosciences, Franklin Lakes, NJ) followed by incubation with fluorescent antibodies. Cells were washed with 1X PBS, and analyzed in 1X PBS solution.

### Western blotting and immunoprecipitation

Western blotting was performed as previously described^51^. Briefly, uninfected and HIV-1 infected or untreated and Meth treated CD4^+^ T-cells (after incubation period) were collected in cell lysis buffer, protein lysates were separated on NuPAGE precast gels (Life Technologies Corp.), transferred to 0.45 μm nitrocellulose membranes (Bio-Rad Laboratories, Hercules, CA), and probed with appropriate primary antibodies followed by incubation with their respective secondary antibodies. Proteins were visualized with Western Lightning Plus ECL Substrate (PerkinElmer, Waltham, MA).

For immunoprecipitation assay, CD4^+^ T-cells were left untreated or treated with Meth (100 μM) and incubated for times indicated. Cells were lysed with cell lysis buffer (Cell Signaling Technology). Cell lysates were subjected to immunoprecipitation using Millipore PureProteome^TM^ Protein A and Protein G Magnetic beads, which were used according to manufacturer’s protocol (MilliporeSigma, Burlington, MA) and the immune-complexes were further processed by Western blotting^51^.

### Confocal microscopy

CD4^+^ T-cells were cultured on chamber slides. They were starved for 2 hours, and then untreated or treated with Meth (100 μM) for 24 hours. They were fixed in 4% paraformaldehyde and blocked with 5% normal goat serum in PBS/0.1% Triton X-100 (1 hour). Cells were then incubated with primary antibodies overnight at 4 °C, washed thrice with PBS, and stained with AlexaFluor 488–labeled anti–rabbit IgG antibody and AlexaFluor 594–labeled anti–mouse IgG antibody (Molecular Probes®; Invitrogen) for 2 hours. Subsequently, cells were washed thrice with PBS, and slides were mounted using Prolong Gold antifade with DAPI (4′,6-diamidino-2-phenylindole; Invitrogen). Slides were examined under a Zeiss 880 Meta confocal microscope (Carl Zeiss Microimaging, LLC, Thornwood, NY), and images were acquired using ZEN2 software (Carl Zeiss). Figures were made using Adobe Photoshop CS4 software (Adobe Systems, San Jose, CA).

### Calcium Flux Measurement

CD4^+^ T-cells were labeled with Fluo-4, AM (Invitrogen; Carlsbad, CA) according to the manufacturer’s protocol. Live cell microscopy was performed and fluorescence was measured before and after addition of Meth. Pixel density of the fluorescence was determined using Image J software.

### Quantitative RT-PCR

RNA was isolated from CD4^+^ T-cells using TRIzol™ reagent according to the manufacturer’s instructions (Thermo Fisher Scientific, Waltham, MA). DNase treatment was performed using TURBO DNA-free kit (Ambion RNA, Carlsbad, CA). 1ug of RNA was used to prepare cDNA using iSCRIPT cDNA synthesis kit (Bio-Rad, Hercules, CA). qRT-PCR was done in triplicate for each sample with SYBR green based PowerUp™ SYBR™ Green Master Mix (Thermo Fisher Scientific, Waltham, MA) using 100 ng cDNA. Gene expression was normalized to TATA-box binding protein (TBP) and fold change in expression was calculated using 2−ΔΔCt method. Specificity of the primer sets was confirmed by melting curve analysis. Primer sequences used were:

miR-34c (RefSeq:NR_029840):

RT_SL:GTCGTATCCAGTGCAGGGTCCGAGGTATTCGCACTGGATACGACGCAATC

F: GCGGCGGAGGCAGTGTAGTTAGCT

miR-155(RefSeq:NR_030784): RT_SL:GTCGTATCCAGTGCAGGGTCCGAGGTATTCGCACTGGATACGACACCCCT F:GCGGCGGTTAATGCTAATCGTGAT

IL4 (RefSeq:NG_023252.1):

F:TTTGCTGCCTCCAAGAACAC

R:AATCGGATCAGCTGCTTGTG

IL10 (RefSeq:NG_012088):

F:ACATCAAGGCGCATGTGAAC

R: ACGGCCTTGCTCTTGTTTTC.

### Cyclic AMP ELISA

CD4^+^ T-cells were harvested after treating with Meth (100 μM) for 0, 15, 30 and 60 mins and ELISA was performed for measuring cyclic-AMP levels according to the manufacturer’s instructions (Cayman Chemical, Ann Harbor, MI).

### Statistical Analysis

All experiments were performed in triplicate. Differences between untreated and Meth treated samples were calculated using a standard 2-tailed Student’s t-test. P-values ≤ 0.05 were considered statistically significant. For comparisons involving both Meth and the Sigma-1 inhibitor, one way ANOVA was performed and p-values ≤ 0.05 were considered statistically significant.

## Electronic supplementary material

Dataset 1
