# Supplementary material for: Methamphetamine functions as a novel CD4+ T-cell activator via the sigma-1 receptor to enhance HIV-1 infection
Source: Sci Rep. 2019 Jan 30;9:958. doi: 10.1038/s41598-018-35757-x (PMC6353873; doi:10.1038/s41598-018-35757-x)
Supplement: Supplementary file 1 — Dataset 1 [file 41598_2018_35757_MOESM1_ESM.pdf]

Methamphetamine functions as a novel CD4+ T-cell activator via the sigma-1 receptor to enhance HIV-1 infection

Anil Prasad[1¶], Rutuja Kulkarni[1¶], Ashutosh Shrivastava[1& #], Shuxian Jiang[1&], Kaycie Lawson[1], Jerome E. Groopman[1*]

Supplementary Figure S1

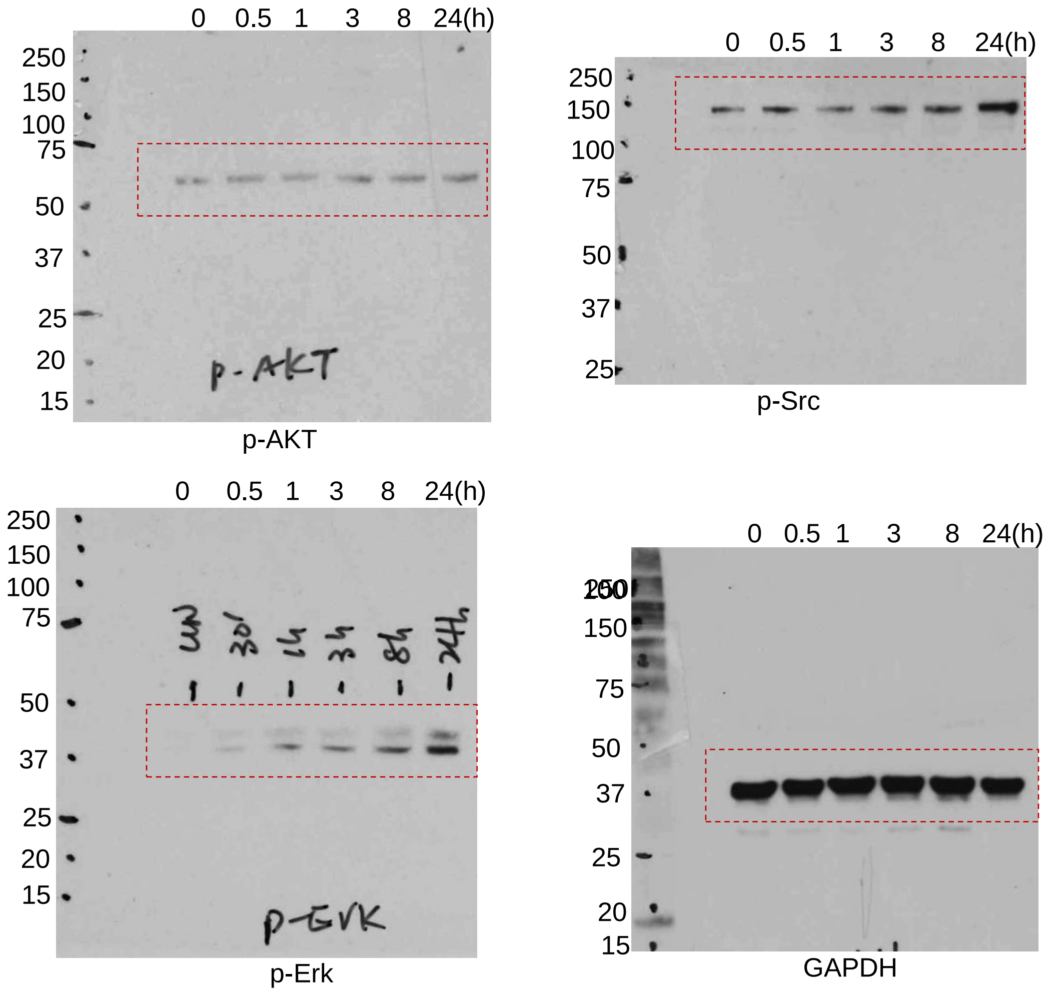

Fig. S1: Full-length blots for Figure 2D

Supplementary Figure S2

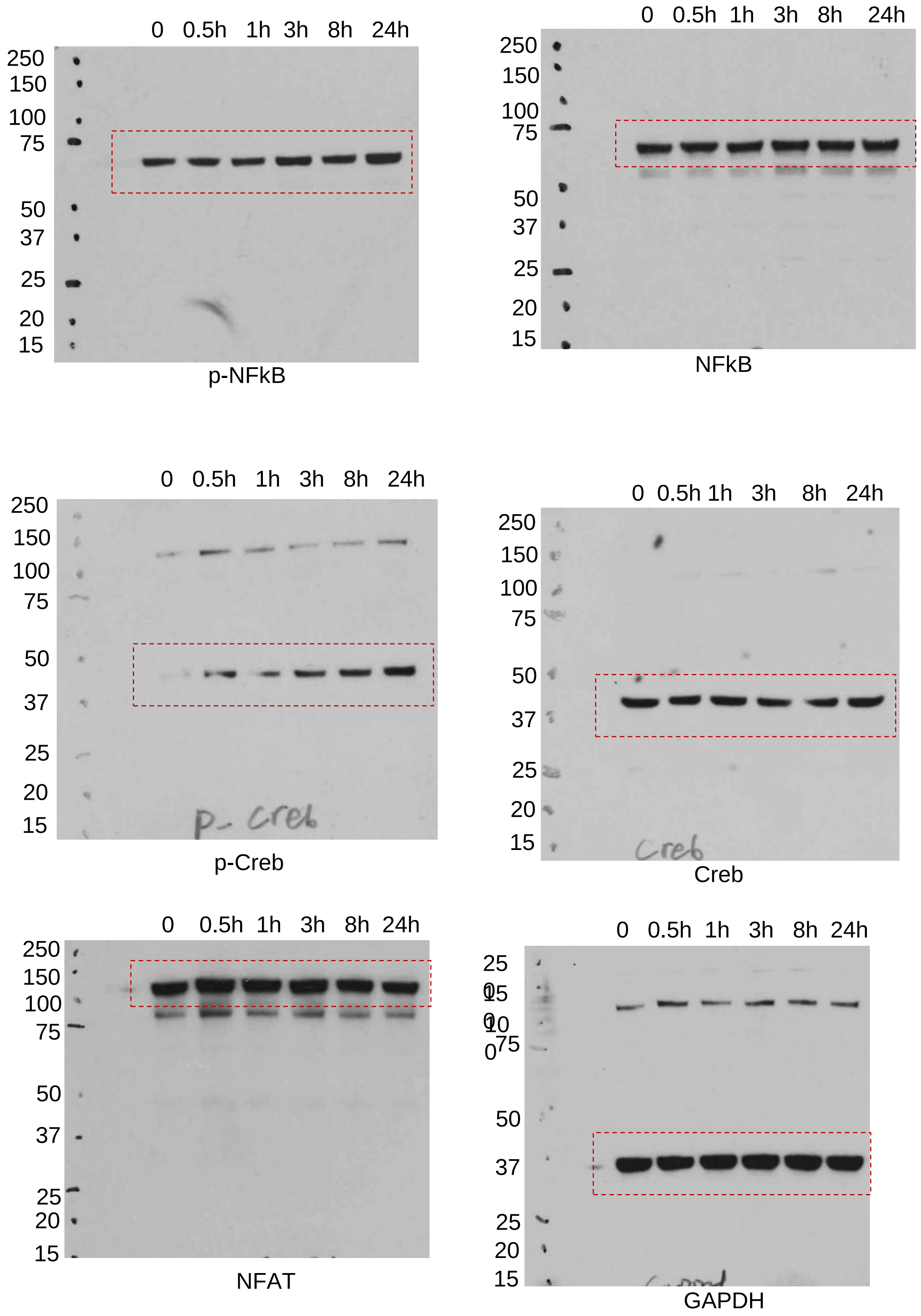

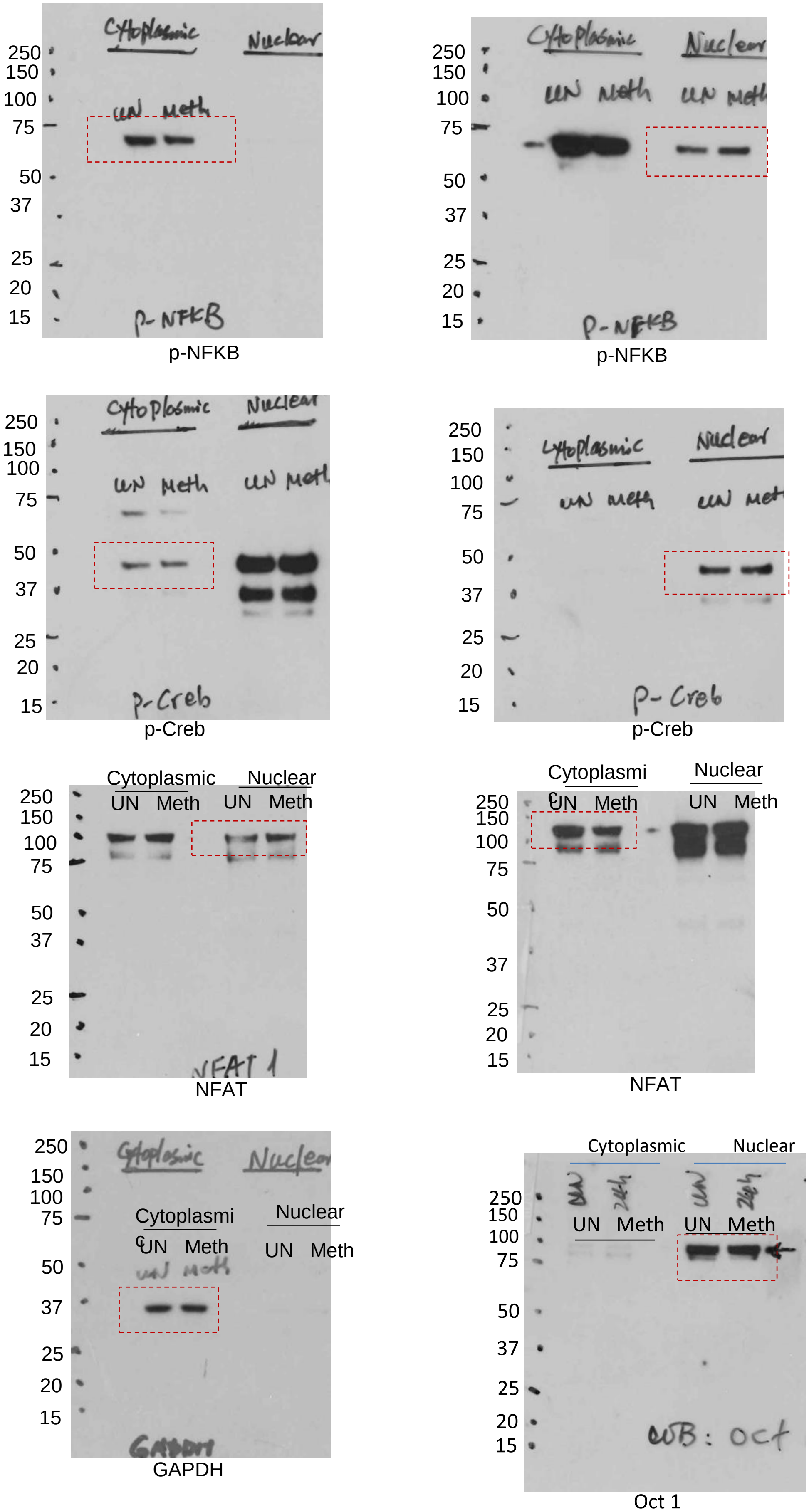

Fig. S2: Full-length blots for Figure 3A and B

Supplementary Figure S3

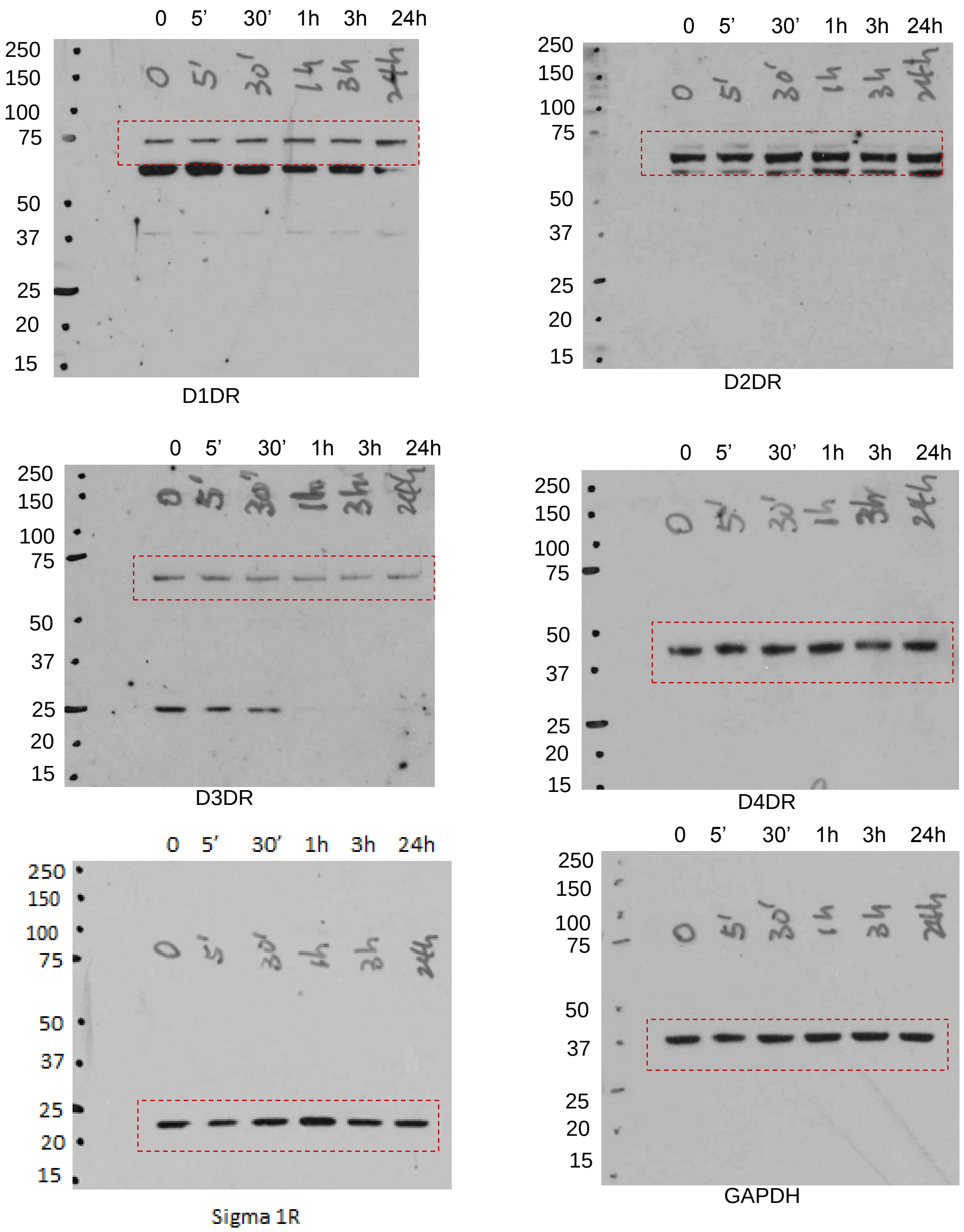

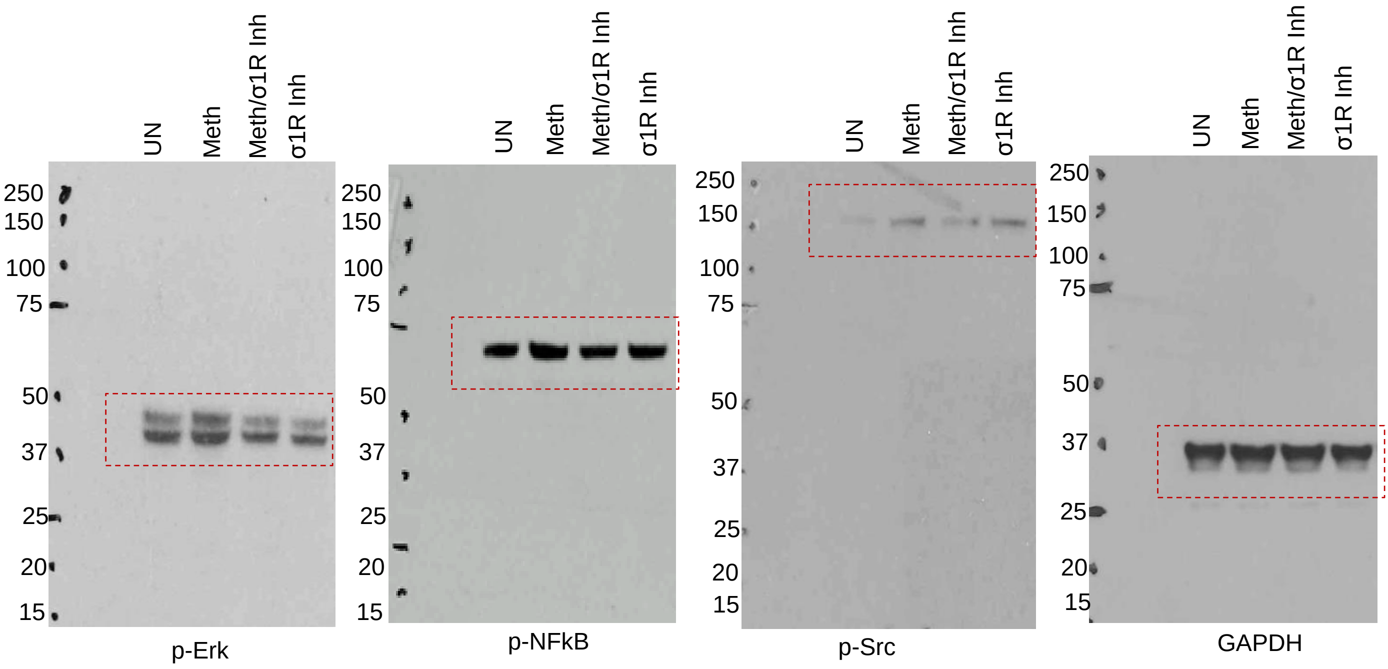

Fig. S3: Full-length blots for Figure 4 A and C

Supplementary Figure S4

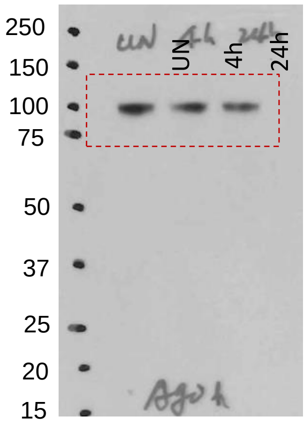

Ago1

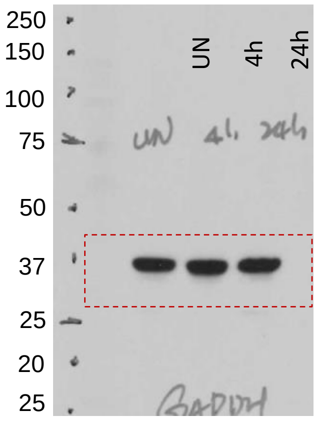

GAPDH

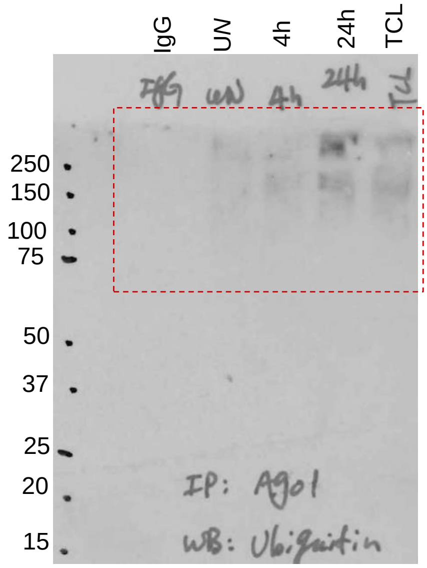

IP: Ago1; WB: Ubiquitin

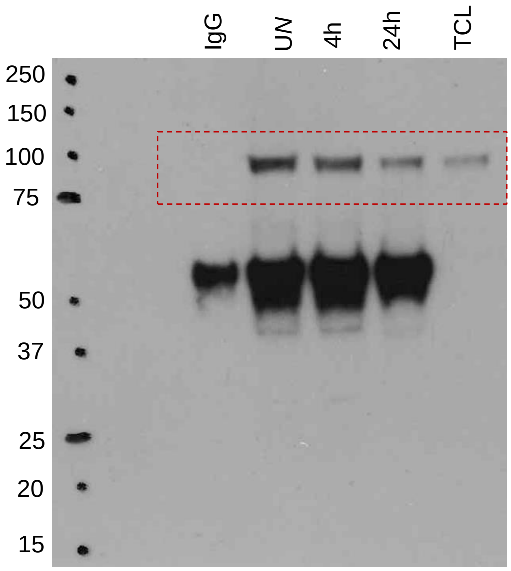

IP: Ago1; WB: Ago1

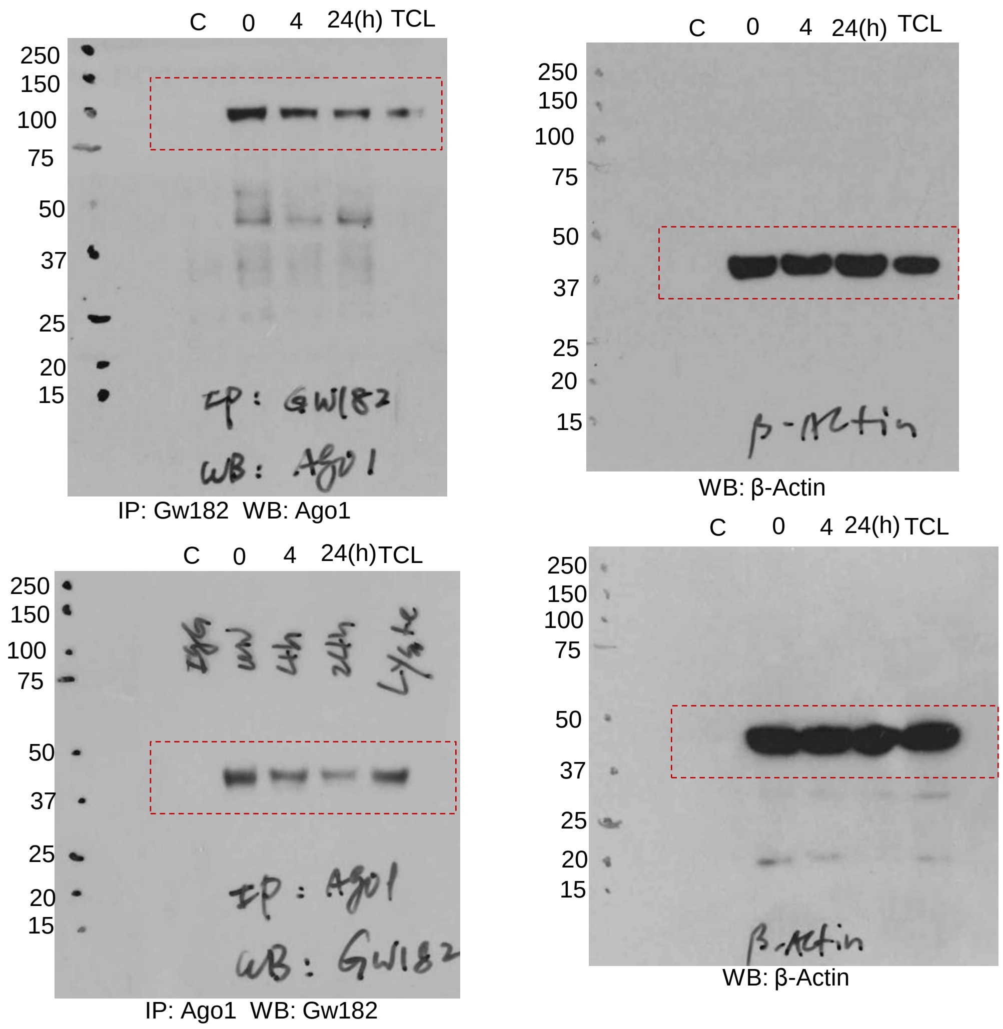

Fig. S4: Full-length blots for Figure 7A-C,
